# Functional Cardiac Tests

**Published:** 1919

**Authors:** 


**Functional Cardiac Tests.** By CAMILLE LIAN. *Annales de Médecine,* September, 1918.

Very many are the soldiers complaining of dyspnea and palpitations following every physical strain, and presenting no cardiac disturbances when examined at rest.

The frequency of such cases has led the author to make the following observations :

(1) Before diagnosing the absence of any cardiac failure, the patient should be submitted to an " effort test ", and examined during, or immediately after, this effort.

(2) In order to appreciate the degree of functional disorders of the heart, the circulatory reactions should be examined after some steady effort accomplished by the patient.

Both the variations in the pulse rate and the arterial pressure should be examined. Other factors than simply the cardiac ones interfere with these symptoms, and in fact their measurement presents great practical importance.

The author's test consists of one minute's double march, without advancing, the legs being flexed at right angles with the thighs, and the patient numbering two steps a second. After one minute, the pulse-rate is measured, the patient standing still.

In normal cases, after this exercise, the pulse-rate is about 120 or 130 a minute; it falls back gradually, after a minute or two at the utmost, to the former frequency, or to some condition quite close to the initial number.

On the contrary, in cases suffering from cardiac disorders, the pulse-rate may rise as high as 160 a minute, and need as long as 5 or 6 minutes to fall back to the former frequency.

A normal result does not always prove, however, that the patient does not suffer from some cardiac disorder. Under certain influences, excitement of the nerves, for instance, an averred cardiac patient may have the same results as a sound subject.

But if an abnormal result is obtained after several examinations, one may be sure that there is some circulatory disturbance, and that the subject is not able to endure a moderate amount of effort.

The author has found the measure of the variations in the pulse-rate of more practical interest and importance than measures concerning arterial pressure.

He suggests also that the conditions in which the "effort test" is undertaken should remain as comparable as possible, since the results might vary with the same subject, according to these different conditions.

